# Novel Isoxazole Derivative Attenuates Ethanol-Induced Gastric Mucosal Injury through Inhibition of H^+^/K^+^-ATPase Pump, Oxidative Stress and Inflammatory Pathways

**DOI:** 10.3390/molecules27165065

**Published:** 2022-08-09

**Authors:** Sidra Razzaq, Amber Mahmood Minhas, Neelum Gul Qazi, Humaira Nadeem, Arif-ullah Khan, Fawad Ali, Syed Shams ul Hassan, Simona Bungau

**Affiliations:** 1 Department of Pharmacology, Riphah Institute of Pharmaceutical Sciences, Riphah International University, Islamabad 46000, Pakistan; 2 Department of Pharmacy, Kohat University of Science and Technology, Kohat 26000, Pakistan; 3 Shanghai Key Laboratory for Molecular Engineering of Chiral Drugs, School of Pharmacy, Shanghai Jiao Tong University, Shanghai 200240, China; 4 Department of Natural Product Chemistry, School of Pharmacy, Shanghai Jiao Tong University, Shanghai 200240, China; 5 Department of Pharmacy, Faculty of Medicine and Pharmacy, University of Oradea, 410028 Oradea, Romania

**Keywords:** gastric ulcer, isoxazole, in-silico, anti-inflammatory, antioxidant, H^+^/K^+^-ATPase inhibition

## Abstract

Isoxazole derivatives are significant enough due to their wide range of pharmacological and therapeutic activities. The purpose of the current study is to use computational, in vitro, in vivo, and extensive molecular approaches to examine the possible anti-ulcer activity of 4-benzylidene-3 methyl-1,2-isoxazol-5(4H)-one (MBO). Biovia Discovery Studio visualizer (DSV) was utilized for virtual screening. A tissue antioxidant investigation, H^+^/K^+^-ATPase test, and anti-H. pylori activities were carried out. ELISA, immunohistochemistry, and PCR methods were employed for the proteome analysis. An ethanol-induced stomach ulcer model was used to examine the anti-ulcer potential in rats. The binding affinities for MBO ranged from −5.4 to −8.2 Kcal/mol. In vitro findings revealed inhibitory activity against H. pylori and the H^+^/K^+^-ATPase pump. It also enhanced levels of glutathione, catalase, and glutathione-S-transferase and reduced lipid peroxidation levels in gastric tissues of rats. In vivo results showed the gastro-protective effect of MBO (30 mg/kg) in ulcerative rat stomachs. The proteomic study revealed decreased expression of inflammatory markers (cyclooxygenase-2, p-NFkB, and TNF-α). In RT-PCR analysis, the expression levels of H^+^/K^+^-ATPase were reduced. Furthermore, ADMET (absorption, distribution, metabolism, excretion and toxicity) studies revealed that MBO has high GIT solubility and has a safer profile for cardiac toxicity. This study suggests that MBO displayed anti-ulcer potential, which may have been mediated through the inhibition of the H^+^/K^+^-ATPase pump, as well as antioxidant and anti-inflammatory pathways. It has the potential to be a lead molecule in the treatment of peptic ulcers with fewer adverse effects.

## 1. Introduction

Ulcers are basically a disruption of the skin or mucus membrane marked by sloughing of inflamed dead tissue. Peptic ulcer disease (PUD) is a chronic injury causing a mucosal break or lesions in the stomach and duodenum, which can also occur anywhere from the pylorus to the cardiac tissue [1]. Mucosal damage due to an ulcer in the stomach is specifically termed a gastric ulcer. In 2017, the reports estimated that circa 10% of the population is affected by PUD, causing 15,000 deaths per year globally [2]. Gastric ulceration occurs when the equilibrium between gastric offensive factors such as pepsin and acid secretion as well as protective mucosal factors, including mucin secretion and antioxidant enzymes, is disturbed [3]. Associated risk factors for PUD are *H. pylori* infection, Zollinger–Ellison syndrome, NSAID use and alcohol consumption [4]. The prevalence of *H. pylori* is around 50% globally; however, the prevalence is higher in developing countries [5].

PUD is characterized by epigastric discomfort, anorexia, nausea, vomiting and bloody stools. Hence, the objective is to alleviate pain, cure the ulceration and prevent relapse. Prevailing therapeutic options are anti-secretory medications, which include proton pump inhibitors (PPIs), histamine (H_2_) receptor antagonists and cytoprotectives, while antimicrobials are preferred in *H. pylori* infection [6,7]. Clinical evaluation of conventional therapies has demonstrated the incidence of drug interactions, various adverse effects in long-term therapy, relapse and increased resistance of *H. pylori* [8]. Thus, the present study emphasizes the development of novel and more effective anti-ulcer medication which can strengthen defense mechanisms, inhibit aggressive factors and prevent ulcer formation.

Isoxazole nucleus belongs to the group of five-membered heterocycles containing oxygen and nitrogen atoms. The unsaturated property of the molecule is contributed by two carbon-carbon double bonds. It is a potent pharmacophore that occurs naturally as ibotenic acid and is also present in a number of marketed drugs with diverse therapeutic activities, such as valdecoxib, risperidone, cycloserine, dicloxacillin, sulfisoxazole, leflunomide and danazol [8]. Due to its relatively easy synthesis, the isoxazole ring has been an object of interest for chemists and pharmacologists from research groups all over the world. Its chemical modifications include both connection of isoxazole with other aromatic, heteroaromatic or non-aromatic rings and substitution with different alkyl groups. Pharmacological activities of substituted isoxazoles, such as antimicrobial, analgesic, antioxidant activity, anti-inflammatory and anticancer activities, have been reported [9].

**Figure 1:**
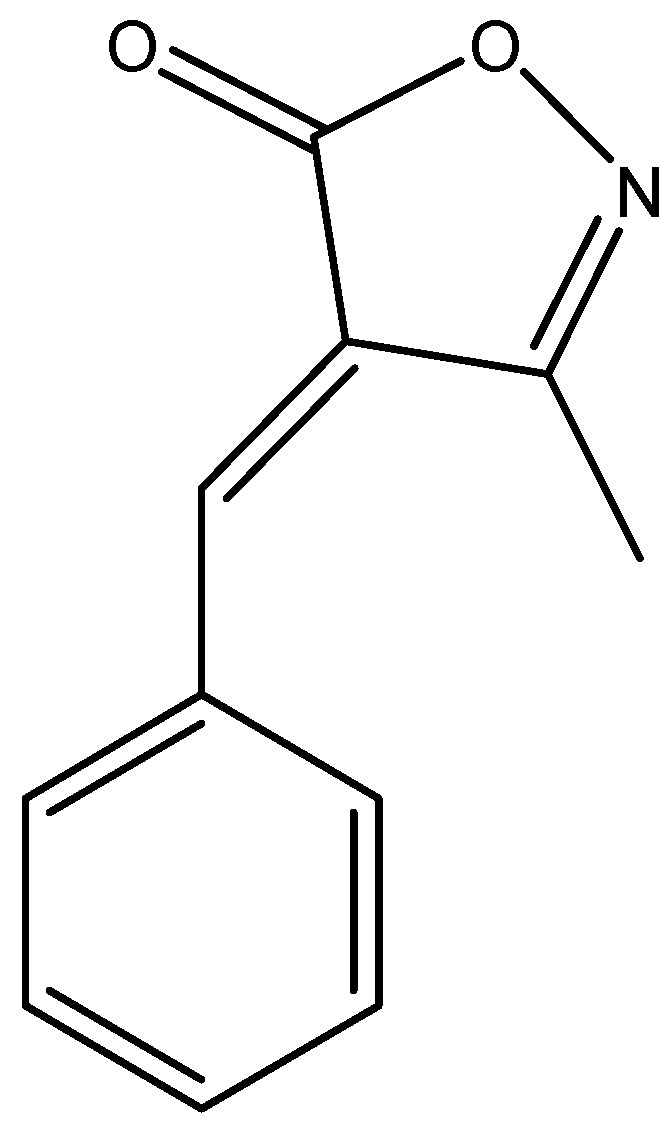
Chemical structure of 4-benzylidene-3 methyl-1,2-isoxazol-5(4H)-one (MBO).

An adequate number of anti-ulcer therapies are available in practice that have brought significant improvements to the ulcerative patient’s life. However, there is always a need for the development of safer drugs. Thus, the goal of the present research is to explore the anti-ulcer potential of 4-benzylidene-3 methyl-1,2-isoxazol-5(4H)-one by computational, in vitro, in vivo and comprehensive molecular techniques (Figure 1).

## 2. Materials and Methods

### 2.1. Chemicals

Absolute ethanol was procured from Sigma Chemicals Co., Ltd. (St. Louis, MO, USA). Omeprazole was obtained from a local pharmaceutical company. Avidin-biotin Complex (ABC) and primary antibodies, including rat anti-TNF-α (SC-52B83), rat monoclonal anti-p-NFkB (SC-271908) and rat anti-COX-2 (SC-514489), were acquired from Santa Cruz Biotechnology (Dallas, TX, USA). 3,3-diaminobenzidine peroxidase (DAB), 5,5′-dithiobis (2-nitrobenzoic acid); (DTNB), GSH, and 1-chloro-2,4-dinitrobenzene (CDNB) were acquired from Sigma Aldrich (St. Louis, MO, USA). Secondary antibody was obtained from Abcam (ab-6789, Cambridge, UK), and proteinase K was purchased from MP Bio, USA. H^+^/K^+^-ATPase activity assay screening kit (Catalog No: E-BC-K122-S), Rat p-NFkB ELISA kit (Catalog No: E-EL-RO674), and rat TNF-α ELISA kit (Catalog No: E-EL-R0019) were procured from Elabscience Biotechnology (Wuhan, China). Protein assay kit BCA (Catalog No: 23227, Waltham, MA, USA) was also used. All other reagents utilized were of analytical grade.

#### Chemical Information of MBO

Yellow crystal: mp 140–142 °C (Lit [2k] mp 142–144 °C); 1 H-NMR (300 MHz, CDCl3): d 2.31 (s, 3H, CH3), 7.44 (s, 1H, ArCH=), 7.49–7.59 (m, 3H, Ar), 8.35 (dd, *J* = 1.3, 7.4 Hz, 2H, Ar); 13C-NMR (300 MHz, CDCl3): d 11.63 (CH3), 119.65 (C=, inside of isoxazolone ring), 129.03 (Ar), 130.47 (Ar), 132.29 (Ar), 134.01 (Ar), 149.98 (ArCH=), 161.16 (C=N), 167.88 (C=O); IR (KBr) n: 1732 (C=O), 1620, 1100, 1216, 879, 763 cm^−1^ [9].

### 2.2. Animals

Sprague-Dawley rats obtained from Laboratory Animal Research Center, Riphah Institute of Pharmaceutical Sciences (RIPS), Islamabad, Pakistan, both male and female, weighing 150–200 g and kept in standard enclosures with a controlled environment (20–25 °C), were fed ad libitum. All experimental procedures were conducted in combination with the Laboratory Animal Resources, Commission on Life Sciences, National Research Council [10] and endorsed by the ethics committee of Riphah Institute of Pharmaceutical Sciences (Ref No: REC/RIPS/2021/003) (Islamabad, Pakistan).

### 2.3. In Silico Studies

To investigate ligand-protein affinity, the Autodock Vina program was used. Additionally, 3D structures of protein targets were acquired from protein data bank RCSB PDB (http://www.rcsb.org/pdb/, accessed on 1 June 2022). Protein targets involved in gastric ulcer pathophysiology include H^+^/K^+^-ATPase (PDB ID: 5ylu), muscarinic M1 (PDB ID: 5CXV), histaminergic H2 (PDB ID: 7ul3), COX-1 (PDB ID: 6y3c), COX-2 (PDB ID: 5f1a), TNF-α (PDB ID: 4TSV) and NFkB (PDB ID: 1A3Q) [11]. These protein targets were prepared via AutoDockTools (version 1.5.6, Scripps Research, San Diego, CA, USA); water molecules were removed, whereas polar H-atoms and charges were added. Structures of standard drug molecules were downloaded from the PubChem database (https://pubchem.ncbi.nlm.nih.gov/search/, accessed on 1 June 2022). Reference drugs used were omeprazole (PubChem CID 4594), pirenzepine (PubChem CID 4848), ranitidine (PubChem CID 3001055), aspirin (PubChem CID 2244) and curcumin (PubChem CID 969516). These structures were downloaded in xml format and converted to PDB format via Open Babel JUI software (Open Babel development team, Cambridge, UK). The 3D structure of MBO was drawn in PDB format through Biovia Discovery Studio Visualizer Client 2016 (DSV v16.1.0.15350). The PDB formats of target proteins, standard drugs and ligand molecules were converted to PDBQT format via AutoDockTools (version 1.5.6). Molecular docking was carried out via Pyrx 0.8 and selecting Autodock Vina as docking software [12,13]. The results were analyzed as the binding affinities/E-values (kcal/mol) and best binding pose. Post docking analysis via Biovia Discovery Studio Visualizer Client 2016 (DSV v16.1.0.15350) was carried out using the one best pose with the lowest energy value [14]. Furthermore, 2D and 3D images were evaluated to determine ligand and amino acid residue interactions.

### 2.4. Anti H. pylori Activity

The antibacterial potential of MBO against *H. pylori* was assessed using the disc diffusion technique. Strains of *H. pylori* in triplicate were obtained from gastric biopsies of gastric ulcer patients with consent at the Care Endoscopy Clinics and Labs (Rawalpindi, Pakistan). They were identified by microaerophilic growth (at 37 °C), colony morphology and urease tests. Sterile McCartney bottles were used to store isolates at −80 °C. Metronidazole served as a positive control. The diameter of the zone of inhibition surrounding each disc was measured and reported as the mean of inhibition diameters (mm) on treated plates incubated for 3–5 days at 37 °C [15].

### 2.5. H^+^/K^+^-ATPase Inhibitory Assay

The inhibitory effect of MBO on gastric H^+^/K^+^-ATPase of rats was analyzed by using the colorimetric method [16]. The commercially available colorimetric H^+^/K^+^-ATPase activity assay screening kit (Catalog No: E-BC-K122-S; Elabscience) was used for analysis. Stomach tissues kept in a bio-freezer (−80 °C) were homogenized and then subjected to centrifugation at 3500 rpm for 10–15 min to obtain a supernatant. The supernatant collected was then analyzed for the release of inorganic phosphate after ATP hydrolysis spectrophotometrically at 660 nm. Here, 1 ATPase activity unit is defined as 1 µmol of inorganic phosphorus released by ATP hydrolysis by ATPase of 1 mg of tissue protein per hour. Findings were then expressed as µmol Pi/mg prot/h.

### 2.6. Antioxidant Profile

The isolated gastric tissue of rats was subjected to homogenization and then centrifuged at 1500 rpm for 30 min to collect the supernatant. The collected supernatant was analyzed for glutathione (GSH), glutathione-S-transferase (GST), catalase and lipid peroxidation (LPO) content assessment. The final product of DTNP and GSH oxidation is 2-nitro-5-thiobenzoic acid, which is yellow in colour. At 412 nm, absorbance was measured using a microplate reader. GSH values were expressed as μmoles/mg of proteins. The formation of CDNB conjugate determined the GST level, and absorbance was measured at 340 nm. It was expressed as μmoles of CDNB conjugate/min/mg of proteins. In the presence of catalase, degradation of H_2_O_2_ was measured using a microplate reader at 240 nm absorbance, and values of catalase were expressed as μmoles H_2_O_2_/min/mg of proteins. Levels of LPO were measured by the assessment of the MDA (malondialdehyde) end product. Absorbance was determined at 532 nm and expressed in TBARS nmoles/min/mg of proteins [17,18].

### 2.7. Ethanol-Induced Gastric Ulcer Model

For induction of gastric lesions, fasted rats (24 h) were randomly assigned to different groups, with five animals in each group. As a negative control, Group I was given saline (10 mL/kg body weight). Groups II, III, and IV were administered MBO at dosages of 5, 10, and 30 mg/kg (p.o.), whereas Group V was given omeprazole (30 mg/kg) as a positive control. One hour after all treatments, absolute ethanol (1 mL/100 g) was given orally to each rat to induce gastric ulcers. All rats were sacrificed by cervical dislocation after one hour of ethanol administration. Stomachs were cleansed with normal saline after removal, and ulcer index in mm was calculated by assessing all lesions. Every lesion’s surface area was calculated and scored according to the previously described method by [19]. The ulcer index (UI) was calculated using the sum of the length (mm) of all stomach lesions. The following formula was used to compute the percentage of inhibition (% I):

% I = (USc − USt) × 100/USc

where USt represents the ulcer surface area of the test group, and USc represents the ulcer surface area of the control group.

The data were analyzed using one-way ANOVA followed by Tukey’s posthoc test. For molecular studies, gastric tissues were preserved at −80 °C in a bio-freezer.

### 2.8. Hematoxylin and Eosin (H&E) Staining

Absolute xylene (100%) was used for de-paraffinization of stomach tissue slides, and then rehydration was accomplished by the use of ethanol (absolute) and ethanol dilutions ranging from 100% to 70%. Subsequently, PBS was used for washing slides, and slides were then placed in hematoxylin for 10 min. Furthermore, slides were then treated for 5 min with 1% HCl solution and ammonia water and then washed with water. After that, for 5–10 min, eosin was applied to slides, washed with water and then dried off. Subsequently, 70%, 95%, and 100% serial dilutions of ethanol were used for dehydration of slides, and then after fixation in xylene, coverslips were placed [20]. Microscopic images were taken using an Olympus light microscope (Olympus, Tokyo, Japan). Images were then analyzed by Image J NIH (Wayne Rasband, Kensington, MA, USA). A total of 5 microscopic images from each group were taken and analyzed for stomach tissue morphology, vacuolation and cellular necrosis [21].

### 2.9. Immunohistochemistry (IHC) Investigation

Deparaffinized stomach tissue slides were treated with proteinase K (enzymatic technique) for antigen retrieval before being rinsed with PBS. Endogenous peroxidase activity was inhibited by immersing slides in 3% H_2_O_2_ for 10 min. Normal goat serum (5% NGS with 0.1% Triton X-100) was applied to slides after rinsing with PBS and incubated for at least 1 h. Slides were treated with primary antibodies including rat anti-COX-2, rat anti-TNF-α, and rat anti-p-NFkB (Dilution 1:100) and incubated at 4 °C overnight. The slides were rinsed in PBS the next day, then treated with a biotinylated secondary antibody (dilution factor 1:50) and again incubated for 1.5 h. After another PBS wash, the slides were incubated in a humidified chamber for 1 h with ABC. In the last phase, staining of slides was carried out by immersion in DAB chromogen. Slides were then rinsed in distilled water and with ethanol for dehydration, followed by xylene fixation, and coverslips were placed after applying mounting media. Immunohistochemical images (three images on each slide) were taken with a microscope (Olympus, Japan) and saved in TIF format. COX-2, TNF-α and p-NFkB expression was determined using Image J software (Wayne Rasband, Kensington, MA, United States). According to the threshold intensity, the background of images was optimized, and COX-2-, TNF-α- and p-NFkB-positive cells were analyzed and represented as the relative integrated density of the samples in comparison to saline [22].

### 2.10. Enzyme-Linked Immunosorbent Assay (ELISA)

In accordance with the manufacturer’s instructions (Elabscience, Wuhan, China), TNF-α (Cat. #: E-EL-R0019) and p-NFkB (Cat. #: E-EL-RO674) expression was determined. Stomach tissues (50 mg) kept in a bio-freezer (−80 °C) were subjected to homogenization at 15 rpm × 1000 using SilentCrusher M (Heidolph, Schwabach, Germany) before being centrifuged at 1350× *g* for 1 h. A supernatant was obtained. Total protein content was determined by the BCA (bicinchonic acid) method. Using a 96-well plate in a kit, samples were processed with specified antibodies, and absorbance was measured using a microplate reader. Concentrations (pg/mL) were then normalized to total protein content in picograms per milligram (pg/mg total protein), and the experiment was repeated at least three times. For analysis, one-way ANOVA was performed, followed by Tukey’s posthoc test [23,24].

### 2.11. Real-Time Polymerase Chain Reaction (RT-PCR) Analysis

Following the homogenization of gastric tissues, the trizol method was used to extract total ribonucleic acid (RNA) as specified by the manufacturer. From total RNA (1–2 µg), cDNA was synthesized by reverse transcriptase enzyme, and cDNA was then amplified by real-time PCR using a thermocycler [25]. The mRNA expression was normalized to expression levels of beta-actin. For real-time quantitative PCR, the relative gene expression was calculated using the 2^ΔΔ^-CT technique. Two primer sets, forward and reverse, were used to enhance the annealing temperature, as shown in Table 1.

### 2.12. ADMET Analysis

ADMET (absorption, distribution, metabolism, excretion, and toxicity) are the essential measurement tools for any compound before being elected as a drug candidate. The online web tool swiss ADME (http://www.swissadme.ch/index.php, accessed on 1 June 2022) was used to obtain ADME properties of the isolated flavone [26], and the pharmacokinetic scores were predicted using the online web application pkCSM (http://biosig.unimelb.edu.au/pkcsm/prediction, accessed on 1 June 2022).

### 2.13. Cardiac Toxicity

The blockage of the hERG channels is linked to fatal cardiac arrhythmias. The pre-hERG 4.2 (http://predherg.labmol.com.br/predict, accessed on 1 June 2022), a web tool, was used for early predictive cardiac toxicity.

### 2.14. Statistical Analysis

Through Image J software, morphological data were analyzed. The data were presented as mean SEM (*n* = 5). The findings were analyzed statistically using one-way ANOVA, followed by a posthoc Tukey’s test using the Graph Pad program (GraphPAD, San Diego, CA, USA). A *p*-value of less than 0.05 (*p* < 0.05) was regarded as significant.

## 3. Results

### 3.1. In Silico Analysis

Against different target proteins, MBO exhibited variable ACE (atomic contact energy) values. MBO against H^+^/K^+^-ATPase pump, M1, H_2_, COX-1, COX-2, TNF-α and NFkB exhibited an E-value of −7.4 Kcal/mol, −8.2 Kcal/mol, −6.2 Kcal/mol, −7.5 Kcal/mol, −7.7 Kcal/mol, −5.4 Kcal/mol, and −5.7 Kcal/mol respectively. Omeprazole against the H^+^/K^+^-ATPase pump exhibited an energy value of −8.2 Kcal/mol. Pirenzepine against M1 exhibited an energy value of −8.7 Kcal/mol. Ranitidine against H_2_ exhibited an energy value of −5.3 Kcal/mol. Aspirin against COX-1, COX-2 and TNF-α exhibited E-values of −6.7 Kcal/mol, −6.8 Kcal/mol, and −4.9 Kcal/mol, respectively. Curcumin against NFkB exhibited an E-value of −5.8 Kcal/mol. Table 2 summarizes the E-values (kcal/mol), hydrogen bonds, and amino acid residues making H-bonds with the best-docked poses of MBO and standard drugs. The 2D and 3D depictions of MBO and standard drug interactions with their protein targets are shown in Supplementary Figures S1–S14A,B. Standard inhibitors of the pathways are omeprazole, pirenzepine, ranitidine, aspirin, Cox-1, Cox-2 and curcumin. The amino acids are alanine (ALA), aspartic acid (ASP), asparagine (ASN), arginine (ARG), cysteine (CYS), glycine (GLY), glutamine (GLN), glutamic acid (GLU), histidine (HIS), isoleucine (ILE), leucine (LEU), lysine (LYS), proline (PRO), phenylalanine (PHE), serine (SER), tyrosine (TYR), tryptophan (TRP), threonine (THR), and valine (VAL).

### 3.2. Effect of MBO on H. pylori Inhibition

The anti-H. pylori activity of MBO and metronidazole was evaluated in vitro by disc diffusion assay. Table 3 presents the zone of inhibition diameter (mm) at concentrations of 0.5, 1, 2, 4, 8, 16 and 32 µg/disc and MIC50 (µg/mL) values of three clinical strains of H. pylori. MBO shows MIC50 (minimum inhibitory concentration 50, µg/mL) values of 15, 14 and 14 for strains I, II and III, respectively. Metronidazole shows MIC50 (µg/mL) values of 4, 6 and 4 for strains I, II and III respectively.

### 3.3. Effect of MBO on Rat Gastric H^+^/K^+^-ATPase Inhibition

**Figure 2:**
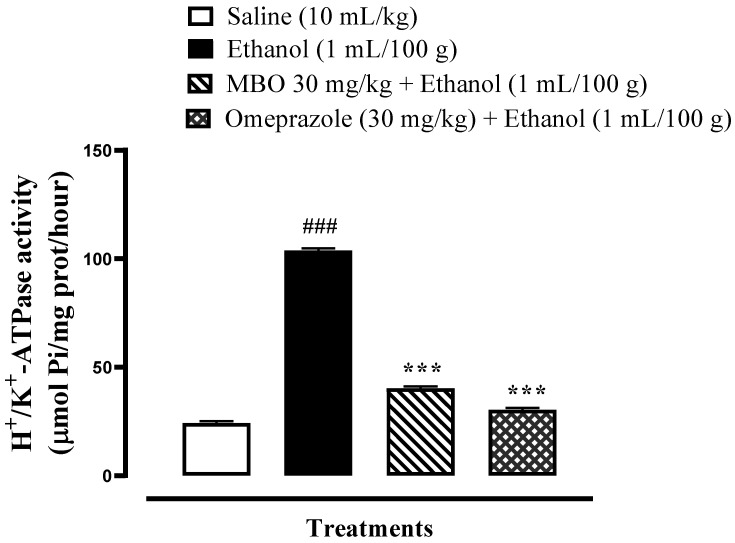
Effect of MBO on rat gastric H^+^/K^+^-ATPase activity. Inhibitory effect was analyzed using the colorimetric method. Values expressed as mean ± SEM (*n* = 3). Significance at *** *p* < 0.001, ^###^ *p* < 0.001.

MBO significantly inhibits the H^+^/K^+^ ATPase comparable to omeprazole (*** *p* < 0.001), as illustrated in Figure 2. MBO inhibits H^+^/K^+^-ATPase enzyme activity at 40.25 µmol Pi/mg prot/h, while omeprazole inhibits the H^+^/K^+^-ATPase activity at 30.29 µmol Pi/mg prot/h.

### 3.4. Effect on Oxidative Stress Markers

**Figure 3:**
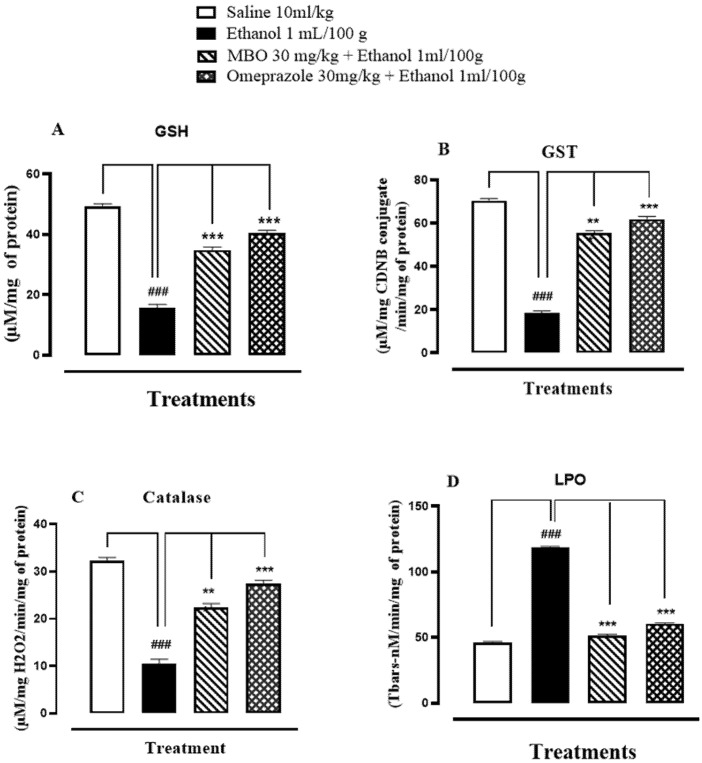
(**A**–**D**) Bar graph representing the effect of MBO and omeprazole against reduced glutathione (GSH), glutathione-s-transferase (GST), catalase and lipid peroxidation (LPO) in ethanol-induced gastric ulcer tissues. Values expressed as mean ± SEM (*n* = 3). One-way ANOVA, with posthoc Tukey’s test. ^###^ *p* < 0.001 vs. saline group, *** *p* < 0.001 vs. ethanol groups.

GSH, GST, catalase and LPO levels in rat stomach tissues were 49.36 + 0.49 μmoles/mg, 70.50 + 0.57 μmoles of CDNB conjugate/min/mg, 32.23 + 0.42 μmoles H_2_O_2_/min/mg, and 46.00 + 0.57 nmoles/min/mg, respectively, in the saline (10 mL/kg) group. In the ethanol (1 mL/100 g) group, GSH, GST, and catalase levels decreased to 15.80 + 0.60 μmoles/mg, 18.66 + 0.46 μmoles of CDNB conjugate/min/mg, and 10.50 + 0.55 μmoles H_2_O_2_/min/mg, respectively, and LPO levels increased to 118.53 + 0.56 nmoles/min/mg. GSH, GST, and catalase levels increased in the MBO (30 mg/kg)-treated group to 34.83 + 0.61 μmoles/mg, 55.5 + 0.60 μmoles of CDNB conjugate/min/mg, and 22.4 + 0.48 μmoles H_2_O_2_/min/mg, respectively, and LPO levels were decreased to 51.60 + 0.51 nmoles/min/mg. In the omeprazole (30 mg/kg) group, GSH, GST, and catalase levels raised to 40.53 + 0.50 μmoles/mg, 61.93 + 0.78 μmoles of CDNB conjugate/min/mg, and 27.5 + 0.37 μmoles H_2_O_2_/min/mg, respectively, and LPO levels reduced to 60.50 + 0.47 nmoles/min/mg. Here, ^###^ *p* < 0.001 versus saline group, *** *p* < 0.001 versus ethanol groups as illustrated in Figure 3A–D.

### 3.5. Effect of MBO on Ethanol-Induced Gastric Ulcer Model

**Figure 4:**
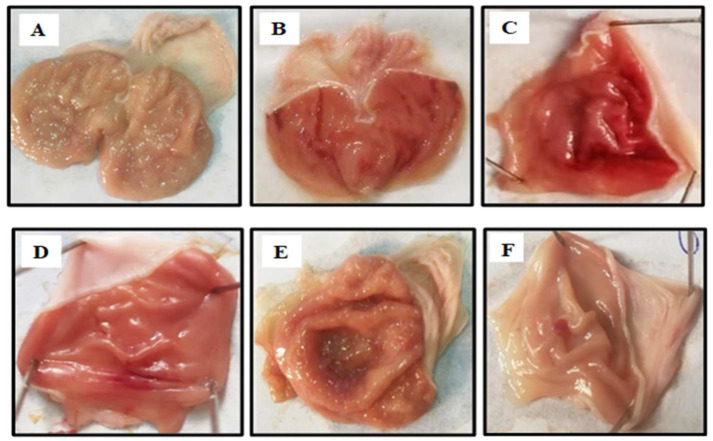
Gross appearance of gastric mucosa in rats: (**A**) treated with saline 10 mL/kg. (**B**) Severe lesions are observed with absolute ethanol (1 mL/100g) treatment along with hemorrhagic necrosis of gastric mucosa. (**C**–**E**): MBO treatment groups at doses of 5 mg/kg, 10 mg/kg and 30 mg/kg, respectively. (**F**) Pre-treated with omeprazole (30 mg/kg). The gastric lesions were reduced with increased doses of MBO and omeprazole compared to ulcer control. At 30 mg/kg, MBO showed the most effective gastro-protective action.

MBO exhibited an anti-ulcer effect in a dose-dependent manner. Table 4 illustrates that oral administration of MBO decreased the absolute ethanol-induced gastric lesions in comparison to the control group. MBO at 5 mg/kg, 10 mg/kg and 30 mg/kg caused 20%, 40% and 90% inhibition, respectively (*** *p* < 0.001 vs ethanol group). Omeprazole (30 mg/kg) induced 90% inhibition. Macroscopic examination revealed the rat’s gastric mucosa, as shown in Figure 4.

### 3.6. Histopathological Examination

**Figure 5:**
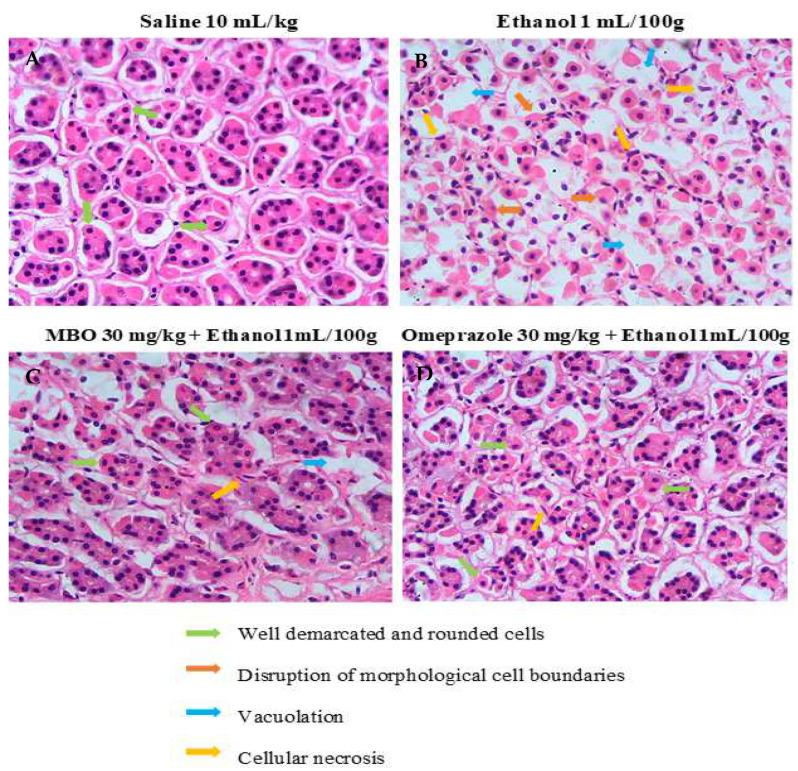
Effect of MBO on ethanol-induced stomach ulcers analyzed by histopathological technique. Gastric tissues (*n* = 5) from each experimental group were processed for histological changes after 1 h of ethanol treatment. Magnification 40×. (**A**) Saline 10 mL/kg; (**B**) ethanol 1 mL/100g; (**C**) MBO 30 mg/kg + ethanol 1 mL/100g; (**D**) omeprazole 30 mg/kg + Ethanol 1 mL/100g. Green, orange, blue, and yellow arrows represent well-demarcated and rounded cells, disruption of morphological cell boundaries, vacoulation and cellular necrosis, respectively.

Figure 5 illustrates the histopathological analysis of the gastric tissues. In H&E staining, gastric cells were analyzed to assess whether they were in a normal state or displayed any changes in cell morphology. The saline group (10 mL/kg) shows normal cell morphology with intact shape and size of gastric cells. The ethanol group (1 mL/100g) shows cellular necrosis and disrupted cell boundaries. In comparison, cell shape, size, boundaries and vacuolation were restored in the MBO (30 mg/kg) and omeprazole (30 mg/kg) treatment groups.

### 3.7. Immunohistochemistry (IHC) Analysis

**Figure 6:**
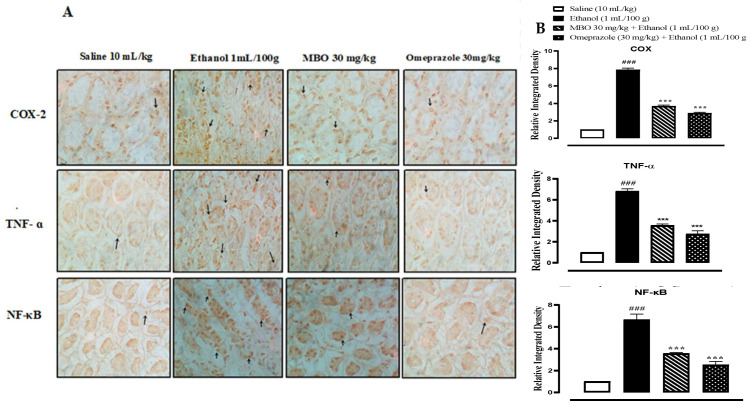
(**A**) represents the inhibitory effect of MBO against COX-2, TNF-ɑ, and p-NFkB expression in gastric tissues of rats using the immunohistochemical technique (Magnification 40×). Saline, ethanol, MBO 30 mg/kg and omeprazole group. (**B**) Graphical representation of severity scores of stomach injury in different groups calculated by relative integrated density. Values expressed as mean ± SEM (*n* = 4). One-way ANOVA with posthoc Tukey’s test. ^###^ *p* < 0.001 vs. saline group, *** *p* < 0.001 vs. ethanol group.

In the ethanol group (1 mL/100g), the expression of COX-2, TNF-α and p-NFkB is increased in gastric tissues (^###^ *p* < 0.001 versus saline group). In the MBO (30 mg/kg) and omeprazole (30 mg/kg) treatment groups, their expression is significantly reduced (*** *p* < 0.001 versus ethanol group), as presented in Figure 6A,B.

### 3.8. Effect of MBO on Inflammatory Markers by ELISA

**Figure 7:**
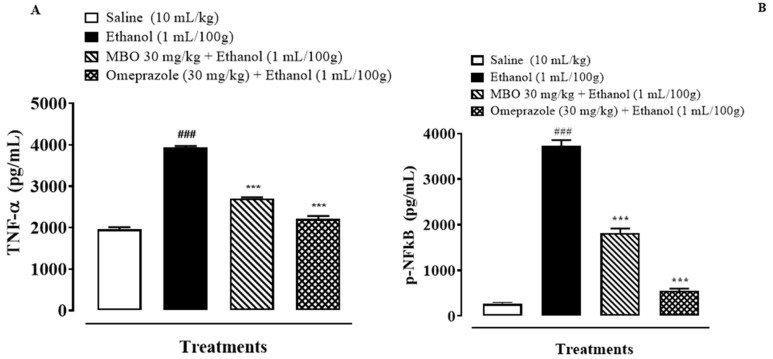
(**A**,**B**) represents effect of MBO on TNF-α and p-NFkB expression in gastric tissue of rats. After 1 h of ethanol treatment, animals were sacrificed; stomachs were isolated and stored at −80 °C. Stomach tissues were homogenized as described in the methods section, and TNF-α and p-NFkB levels were measured by ELISA assay. Values expressed as mean ± SEM (*n* = 3). *** *p* < 0.001; ^###^ *p* < 0.001. Analyzed by one-way ANOVA followed by posthoc Tukey’s test.

Figure 7A,B illustrates the effect of MBO on TNF-α and p-NFkB in the stomach tissues of rats. MBO significantly decreased the TNF-α and p-NFkB expression levels in the treatment group compared to the negative control group (*** *p* < 0.001 versus ethanol group, ^###^ *p* < 0.001 versus saline group). One-way ANOVA was used to evaluate the data, followed by a posthoc Tukey’s test. Isolated stomach tissues from the ethanol-administered group exhibited an increased TNF-α and p-NFkB expression of about 3931.37 + 32.65 and 3732.5 + 100.0 pg/mL of total protein, respectively, compared to the normal saline-treated group. MBO (30 mg/kg) caused a significant decrease in TNF-α and p-NFkB expression of 2705 + 20.41 pg/mL and 1812.5 + 87.77 pg/mL of total protein, respectively, compared to the negative control group. Omeprazole also caused a decrease in the expression of TNF-α and p-NFkB of about 2215 + 53.07 pg/mL and 552.5 + 38.78 pg/mL of total protein, respectively, compared to the negative control group.

### 3.9. Effect of MBO on Expression of H^+^/K^+^-ATPase through RT-PCR Analysis

**Figure 8:**
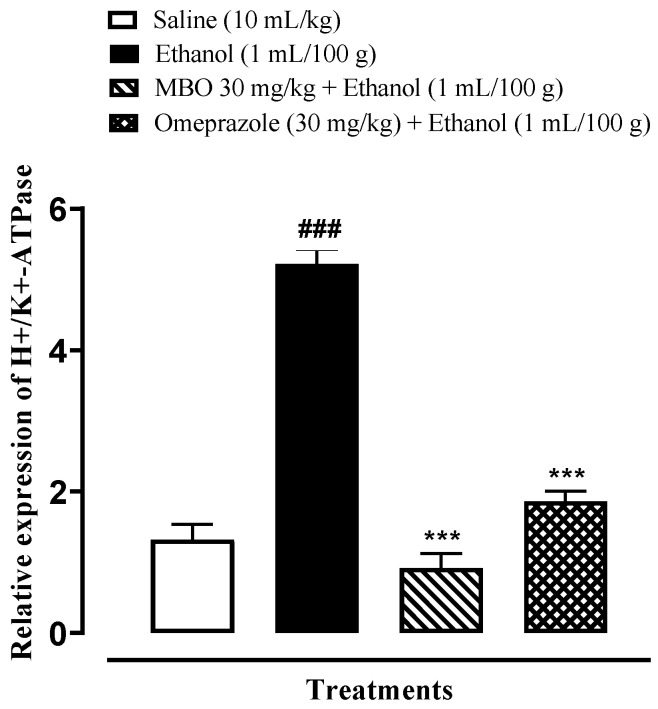
Effects of MBO in H^+^/K^+^-ATPase expression in ethanol-induced gastric ulcer model. The mRNA levels of H^+^/K^+^-ATPase were measured by RT-PCR using specific primers. Significance at *** *p* < 0.001 vs. ethanol group; ^###^ *p* < 0.001 versus saline group. Analyzed by one-way ANOVA followed by posthoc Tukey’s test.

RT-PCR is conducted to demonstrate the expression of H^+^/K^+^-ATPase in an ethanol-induced gastric ulcer model. The H^+^/K^+^-ATPase mRNA levels in the ethanol-administered group were increased compared to the normal saline-treated group. MBO (30 mg/kg) caused a significant decrease in expression levels, whereas omeprazole also reduced the expression compared to the negative control group (*** *p* < 0.001 versus ethanol group, ^###^ *p* < 0.001 versus saline group), as shown in Figure 8. One-way ANOVA was used to evaluate the data, followed by a posthoc Tukey’s test.

### 3.10. Pharmacokinetics and ADMET

**Figure 9:**
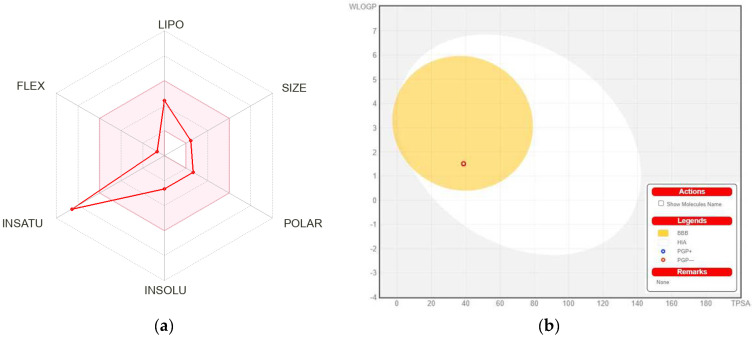
(**a**) Bioavailability radar chart for isolated flavone. The pink zone represents the physicochemical space for oral bioavailability, and the red line represents the oral bioavailability properties. (**b**) Predicted BOILED-Egg plot from *swiss ADME* online web tool for MBO.

The physicochemical and absorption, distribution, metabolism, excretion, and toxicity (ADMET) characteristics of the MBO were deeply investigated and discussed in Table 5. According to Table 5, the MBO showed good results within the limit for lipophilicity, insolubility, size, polarity, and flexibility. The oral bioavailability chart of MBO is mentioned in Figure 9. Among all six factors, only the instauration was out of the limit.

HIA and CNS absorption are essential parameters checked for every drug before its entry for drug formulation in the pharmaceutical field or clinical trials [26,27]. Blood–brain barrier penetration is essential, as compounds that act on the central nervous system (CNS) must cross through the blood–brain barrier, and the compounds that are not active on the CNS should not intersect to avoid adverse effects on the CNS [27]. As mentioned in Table 5, the MBO displayed a high gastrointestinal absorption (HIA) with less BBB permeability, indicating that MBO shows a low occurrence for adverse CNS effects.

Figure 8b shows the BOILED-EGG curve. The BBB penetration and GI absorption (HIA) of the substances may be predicted by this method. There are two areas: one for the GI absorption zone (HIA) and the other for BBB penetration (yolk). Neither GI absorption nor BBB penetration is indicated if any component is found in the gray zone. Furthermore, MBO did not show that it is a P-gp substrate; it is not sensitive to the efflux mechanism of P-gp, which is used by many cancer cell lines to develop resistance to drugs [28], as shown in Figure 8b.

MBO showed 97.1% absorption orally. It has poor penetration into the CNS. MBO only showed inhibition for a specific CYP1A2 cytochrome, which means that MBO can be sensitive to these specific targeted cytochrome drugs. However, for the remaining cytochromes, MBO did not show any inhibition, which shows its safety in the case of drug-drug inhibition. It has a total clearance of 0.662, as mentioned in Table 5. MBO showed hepatotoxicity inhibition, which means that it can cause hepatotoxicity, but for hERG I and hERG II, MBO was totally safe. Skin sensitisation is also an important parameter for many biomolecules [29]. The compound MBO was skin sensitive. In addition, MBO showed no environmental toxicity.

#### Cardiac Toxicity

**Figure 10:**
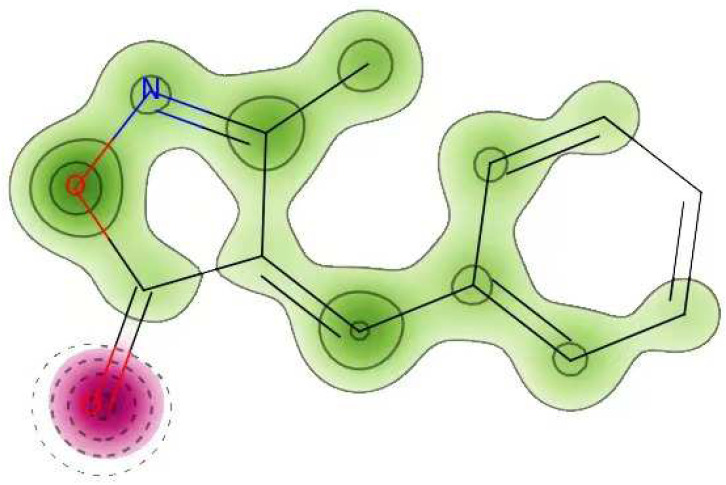
Cardiac toxicity of MBO derived from pred-hERG in a map format.

The FDA requires that every biomolecule be tested for hERG safety before it may be used as a therapeutic candidate. hERG blockage has been connected to deadly cardiac arrhythmias. Using pred-hERG results to predict cardiac toxicity, the likelihood map for MBO is shown here (Figure 10). Attributions to hERG blockage, both positive and negative, are shown in the figure. Increasing the number of contour lines and the intensity of the green color shows that an atom or fragment has made a more positive contribution to the hERG blockage. With a 60% confidence level, the pred-hERG projected that MBO would be non-cardiotoxic. The findings have revealed that MBO is safe for cardiovascular toxicity.

## 4. Discussion

Peptic ulcer disease (PUD) is a global health dilemma. Its etiology is complex and multifactorial, with increased rates of recurrence [30]. Novel approaches are needed to prevent gastric ulceration and hyperacidity and their recurrence [31]. The current study investigates the protective effects of MBO in peptic ulcer disease. The aim of this research study was to explore the anti-ulcer potential of MBO by molecular docking, in vitro and in vivo analysis and confirmation of its pharmacological effects at the molecular level using different molecular approaches.

The affinity of ligands to specific protein receptors is analyzed by molecular docking studies. In silico studies have a key role in drug development and discovery and are used as a primary tool for structure-based evaluation and for indicating specific targets with binding affinity [32]. The ligand MBO was docked in the active pocket of H^+^/K^+^-ATPase, M1, H_2_, COX-1, COX-2, TNF-α and NFkB targets that are likely associated with gastric ulcer pathophysiology. Ligand and protein complex binding affinity is determined by their binding energy values, hydrophobic interactions and hydrogen bonding. The literature reveals that the lower the atomic contact energy value, the higher the binding affinity and the more stable the ligand-protein complex will be [33]. On the basis of energy values against different target proteins involved in gastric ulcer pathophysiology, the order of ligand affinity with the receptors was revealed as TNF-α > NFkB > H/K- > H^+^/K^+^ ATPase > COX-1 > COX-2 > M1 as compared to standard drugs. In this research study, MBO possesses the lowest energy value against TNF-α, suggesting that MBO has maximum binding affinity against TNF-α, which plays a key role in the inflammatory cascade.

*H. pylori* is a lifelong colonizer of the gastric mucosa of humans and is a known etiological factor for the development of a wide spectrum of gastrointestinal diseases, i.e., peptic ulcers, gastric adenocarcinoma, mucosa-associated lymphoid tissue (MALT) lymphoma and chronic gastritis [34]. *H. pylori* disrupts the protective mucous lining of the stomach, penetrates the mucus layer, attaches to the epithelial cells and allows gastric acid to penetrate and cause ulcers. The urease enzyme secreted by *H. pylori* converts urea to ammonia, which contributes to its survival in an acidic environment. An immune response against bacterial proteins can be initiated, which results in chronic inflammation [35]. In this study, the in vitro anti-*H. pylori* activity of MBO was investigated. The MIC50 (µg/mL) values of three isolated strains indicated that it possesses moderate antibacterial activity against *H. pylori*. The findings also revealed that the possible anti-ulcer effect of MBO may be related to a mechanism of *H. pylori* inhibition. Previous studies reported that various antibacterial drugs containing an isoxazole nucleus in their structure, including sulfisoxazole, oxacillin, cloxacillin and sulfmethoxazole, have been marketed for the treatment of infectious diseases [36]. This provides us with evidence that the antibacterial property of the MBO compound is attributable to the isoxazole nucleus.

In this paper, an ethanol-induced gastric ulcer model was used to evaluate the gastro-protective activity of MBO. Ethanol is a gastro-toxic agent which stimulates the H^+^/K^+^-ATPase pump, resulting in the secretion of gastric acids and pepsin. Ethanol-evoked gastric ulcer injury is characterized by epithelial loss, mucosal edema and hemorrhagic lesions with cell necrosis [37]. MBO treatment in the in vivo animal model caused a significant reduction in the ulcer index. A dose-dependent anti-ulcer effect of MBO was determined at 5, 10 and 30 mg/kg, with the highest protection observed at the 30 mg/kg dose.

The detrimental effect of ethanol on the mucosa is mediated by oxidative stress, with the generation of reactive oxygen species (ROS) causing cellular damage in the stomach. There is induction of numerous endogenous antioxidant enzymes for ROS scavenging [38]. Hence, oxidative stress serves a crucial role in the pathogenesis of stomach ulcers. MBO causes a reduction in gastric lipid peroxidation and enhances GSH, GST and catalase levels, which indicates that the anti-ulcerogenic property of MBO may be linked to its antioxidant profile. 

Further, ethanol-evoked ROS are a critical factor that initiate an inflammatory cascade with the release of pro-inflammatory cytokines, which exacerbate inflammation [39]. Monitoring the inflammatory mediators could also be an effective way of inhibiting gastric lesions, so TNF-α, p-NFkB and COX-2 levels were analyzed. ELISA assays were carried out to quantify the protein expression of TNF-α and p-NFkB, which revealed MBO (30 mg/kg) caused a significant decrease in expression. Hence, the protective effect of MBO could be attributed to its anti-inflammatory activity. These findings are further supported by immunohistochemistry analysis of the gastric tissues. MBO protects the gastric tissues by decreasing the expression of inflammatory markers COX-2, TNF-α and p-NFkB. Finally, microscopic examination of gastric tissues in the histological analysis showed improvements in cellular infiltration and cell morphology in the MBO-treated group.

Gastric acid is secreted by the parietal cells present in the stomach. The H^+^/K^+^-ATPase pump in parietal cells transports hydrogen ions in the stomach with the cytoplasmic hydrolysis of ATP. Hyperactivity of the proton pump results in the hypersecretion of acid, which leads to ulcers. The gastric proton pump is a potential target; its inhibition decreases acid production and ultimately heals ulcers [5]. Proton pump inhibitors (PPIs) are used in practice for ulcer management, but their use is limited because of their adverse effects. In this research study, MBO was investigated via an in vitro H^+^/K^+^-ATPase inhibitory assay, which showed that it significantly reduces ATP hydrolysis by the gastric ATPase, hence demonstrating proton pump inhibitory activity similar to the standard drug omeprazole, an irreversible proton pump inhibitor. Additionally, the results of the molecular docking study revealed that MBO exhibited an energy value of −7.4 Kcal/mol, while the standard drug omeprazole an exhibited energy value of −8.2 Kcal/mol against the H^+^/K^+^-ATPase pump. Hence, the anti-ulcer activity of MBO may be due to proton pump inhibition, as revealed by molecular docking and the in vitro inhibitory assay.

RT-PCR is an efficient molecular technique that quantifies a specific region of DNA [30]. RT-PCR analysis was performed for confirmation of the anti-ulcer mechanism of MBO at the molecular level. Findings revealed that H^+^/K^+^-ATPase expression levels in the MBO (30 mg/kg)-treated group were significantly reduced. Hence, RT-PCR analysis verified that MBO exhibits its gastroprotective effect by a proton pump-inhibitory mechanism.

## 5. Conclusions

The research study reveals that 4-benzylidene-3 methyl-1, 2-isoxazol-5(4H)-one (MBO) showed E-values of −5.4 to −8.2 Kcal/mol against particular target proteins involved in gastric pathophysiology. MBO exhibited anti-ulcer potential, mediated probably through H^+^/K^+^-ATPase pump inhibition and antioxidant and anti-inflammatory mechanisms, which demonstrates its therapeutic value in peptic ulcer disease management. These findings provide insight into the development of more suitable and effective anti-ulcer therapy.

## Figures and Tables

**Table 1:** Forward and reverse primer sequences.

Primer Sequences for H^+^/K^+^-ATPase and β-Actin		
Primers	Forward sequence (5′-3′)	Reverse sequence (5′-3′)
Rat Beta-actin	CCCGCGAGTACAACCTTCT	CGTCATCCATGGCGAACT
H^+^/K^+^-ATPase (J02449)	TATGAATTGTACTCAGTGGA	TGGTCTGGTACTTCTGCT

**Table 2:** E-values (Kcal/mol) and post-dock analysis of best pose of MBO and standard drugs with targets.

Target Proteins	MBO			STANDARD DRUGS			
	E-Value (Kcal/mol)	No of H Bonds	Binding Residues Forming H Bonds	Standard	E-Value (Kcal/mol)	No of H Bonds	Binding Residues Forming H Bonds
H^+^/K^+^ ATPase (PDB ID:5ylu)	−7.4	1	SER A:871	Omeprazole	−8.2	3	ALA A:339, CYS A:813, ASP A:137
Muscarinic M1 (PDB ID:5CXV)	−8.2	1	SER A:109	Pirenzepine	−8.7	-	-
Histaminergic H_2_ (PDB ID: 7ul3)	−6.2	-	-	Ranitidine	−5.3	3	ASN A:292, ALA A:232, LYS A:231
Cox-1 (PDB ID:6y3c)	−7.5	1	GLN A:203	Aspirin	−6.7	2	THR A:206, HIS A:207
Cox-2 (PDB ID:5f1a)	−7.7	1	SER A:530	Aspirin	−6.8	2	TYR A:385, VAL A:523
TNF-α (PDB ID:4TSV)	−5.4	1	GLN A:67	Aspirin	−4.9	2	GLY A:68 GLN A:67
NFkB (PDB ID: 1A3Q)	−5.7	1	ARG B:103	Curcumin	−5.8	2	SER B:108, THR B:149

**Table 3:** In vitro antibacterial activity of MBO against three clinical strains of H. pylori using disc-diffusion method (values expressed as mean + SEM (*n* = 3).

	Zone of Inhibition (mm) at Concentrations (µg/disk)							MIC_50_ (µg/mL)
Samples	0.5	1	2	4	8	16	32	
STRAIN I								
MBO	1.66 ± 0.33	2.33 ± 0.33	4 ± 0.57	6.66 ± 0.33	9 ± 0.57	12 ± 0.57	15.33 ± 0.88	15
Metronidazole	3.66 ± 0.33	4.66 ± 0.33	5.33 ± 0.66	7 ± 0.57	10.33 ± 1.20	14.66 ± 0.88	22 ± 1.15	4
STRAIN II								
MBO	1.66 ± 0.33	3 ± 0	4.66 ± 0.33	7.33 ± 0.66	9.66 ± 0.33	13 ± 0.57	16 ± 0.57	14
Metronidazole	4 ± 0.57	5 ± 0.57	5 ± 0.57	7.33 ± 0.88	10.33 ± 1.20	15 ± 1	20.66 ± 1.33	6
STRAIN III								
MBO	2.33 ± 0.33	3.66 ± 0.33	5 ± 0.57	8.33 ± 0.88	9 ± 0.57	13.33 ± 0.88	16.33 ± 0.88	14
Metronidazole	4 ± 0.57	4.66 ± 0.33	5.66 ± 0.88	8 ± 1.15	11.33 ± 0.66	15.66 ± 0.33	22.66 ± 0.66	4

**Table 4:** Protective effect of MBO and omeprazole against ethanol (1 mL/100g)-induced gastric ulcer model in rats.

Treatment	Ulcer Index	% Inhibition
Saline 10 mL/kg	0	100
Ethanol 1 mL/100 g	10 ± 0.1 ^###^	0
MBO (5 mg/kg) + Ethanol 1 mL/100 g	8 ± 0.039 ***	20
MBO (10 mg/kg) + Ethanol 1 mL/100 g	6 ± 0.086 ***	40
MBO (30 mg/kg) + Ethanol 1 mL/100 g	1 ± 0.067 ***	90
Omeprazole (30 mg/kg) + Ethanol 1 mL/100 g	1 ± 0.061 ***	90

Values expressed as mean +SEM (*n* = 5). One-way ANOVA followed by post hoc Tukey’s test. ^###^ *p* < 0.001 saline group, *** *p* < 0.001 vs ethanol group.

**Table 5:** Pharmacokinetic and ADMET values of MBO.

Properties	Parameters	MBO
Physicochemical Properties	MW ^a^ (g/mol)	187.19 g/mol
	Rotatable bonds	1
	HBA ^b^	3
	HBD ^c^	0
	Fraction Csp3	0.09
	TPSA ^d^	38.66
Lipophilicity Log *P_o/w_*	iLOGP	1.95
	XLOGP3	2.23
	MLOGP	1.95
	Consensus	2.15
Absorption	Human intestinal absorption	97.16%
	Caco2 permeability	1.339
	Skin Permeability	−2.466
	P-glycoprotein Substrate	No
Distribution	Blood-brain barrier Permeability	0.382
	CNS permeability	−1.973
Metabolism	CYP3A4 substrate	No
	CYP2D6 substrate	No
	CYP2D6 inhibitor	No
	CYP1A2 inhibitor	Yes
	CYP2C19 inhibitor	No
	CYP3A4 inhibitor	No
Excretion	Total clearance	0.662
	Renal OCT2 substrate	No
Toxicity	Oral rat acute toxicity (LD50)(mol/kg)	2.231
	Oral rat Chronic toxicity (LOAEL) (mg/kg)	2.188
	Hepatotoxicity	Yes
	hERG I Inhibitor	No
	hERG II Inhibitor	No
	AMES toxicity	No
	Max. Tolerated Dose (human) (log mg/kg/day)	0.733
	Fathead Minnow (log mM)	1.108
	*Tetrahymena pyriformis* (log ug/L)	0.902
	Skin sensitisation	Yes

^a^ Molecular weight, ^b^ H-bond acceptor, ^c^ H-bond donor, ^d^ Topological polar surface area.

## Data Availability

All data generated or analyzed during this study are included in this published article and its Supplementary information files.

## Supplementary Materials

The supporting information can be downloaded at: https://www.mdpi.com/article/10.3390/molecules27165065/s1.

## Sample Availability

Samples of the compounds are not available from the authors.
