# Supplementary material for: Novel Isoxazole Derivative Attenuates Ethanol-Induced Gastric Mucosal Injury through Inhibition of H+/K+-ATPase Pump, Oxidative Stress and Inflammatory Pathways
Source: Molecules. 2022 Aug 9;27(16):5065. doi: 10.3390/molecules27165065 (PMC9415046; doi:10.3390/molecules27165065)
Supplement: Supplementary file 1 [file molecules-27-05065-s001.zip › molecules-1816077-supplementary.pdf]

Supplementary Data

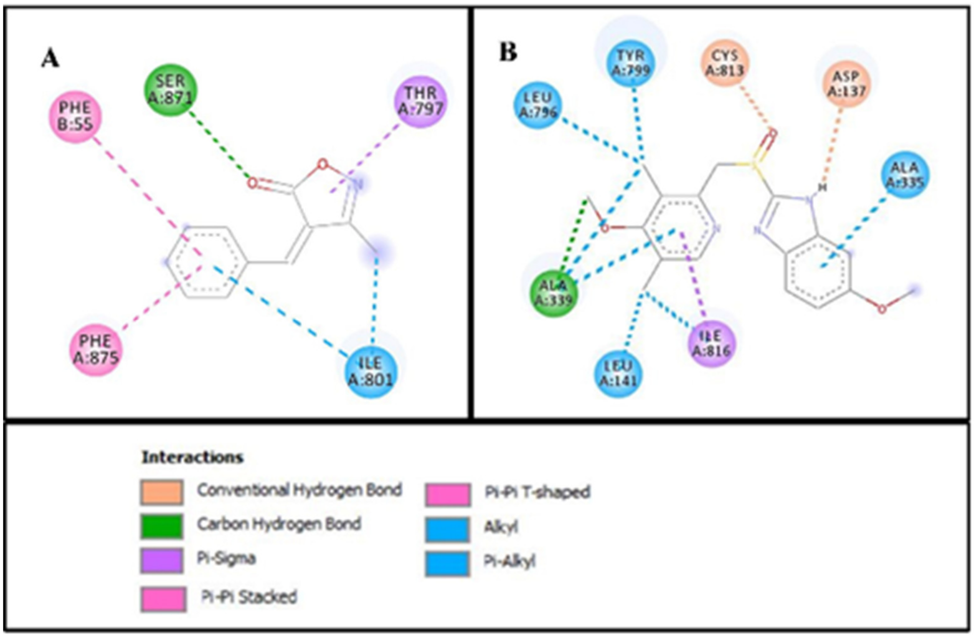

Figure S1. **A** and **B** represents 2D interactions of MBO and Omeprazole against target: H^+/K^+-ATPase respectively, evaluated through Biovia Discovery Studio 2016.

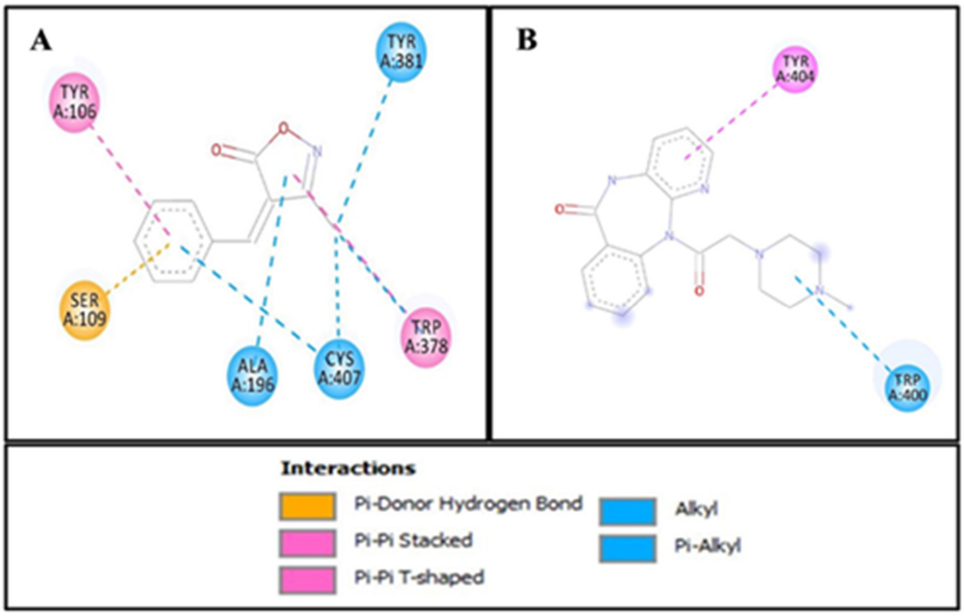

Figure S2. **A** and **B** represents 2D interactions of MBO and Pirenzepine against target: M1 respectively, evaluated through Biovia Discovery Studio 2016.

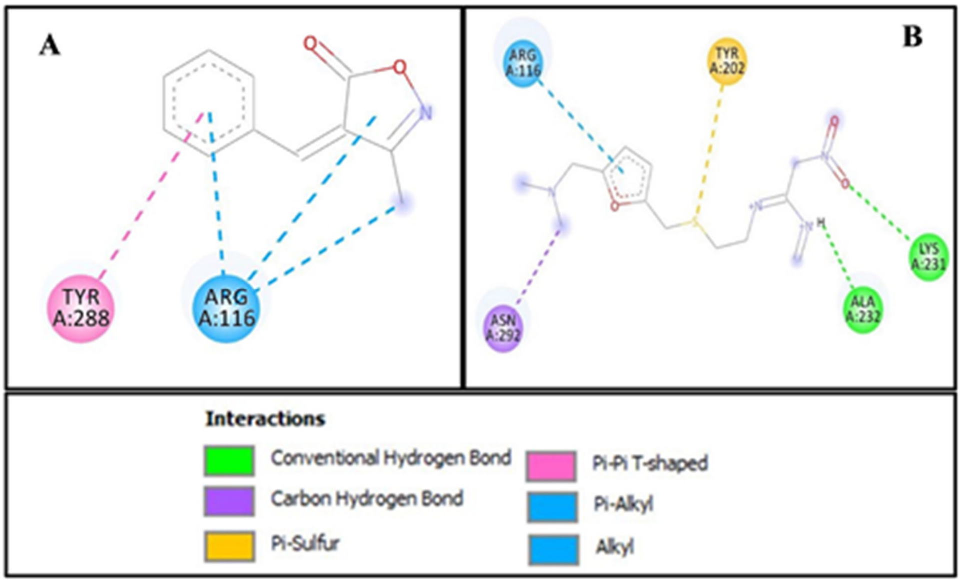

Figure S3. **A** and **B** represents 2D interactions of MBO and Ranitidine against target: H_2 respectively, evaluated through Biovia Discovery Studio 2016.

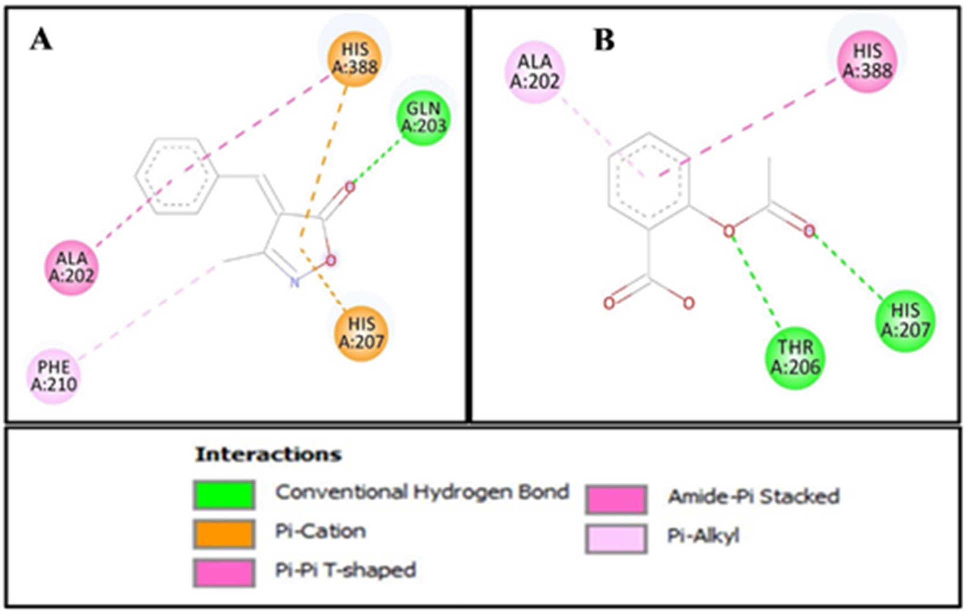

Figure S4. A and **B** represents 2D interactions of MBO and Aspirin against target: COX-1 respectively, evaluated through Biovia Discovery Studio 2016.

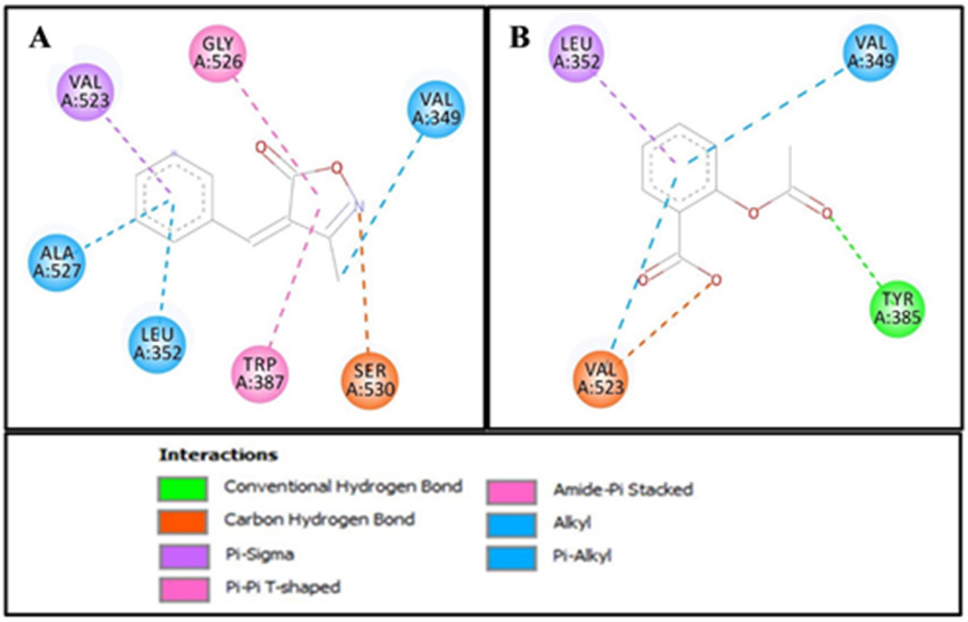

Figure S5. **A** and **B** represents 2D interactions of MBO and Aspirin against target: COX-2 respectively, evaluated through Biovia Discovery Studio 2016.

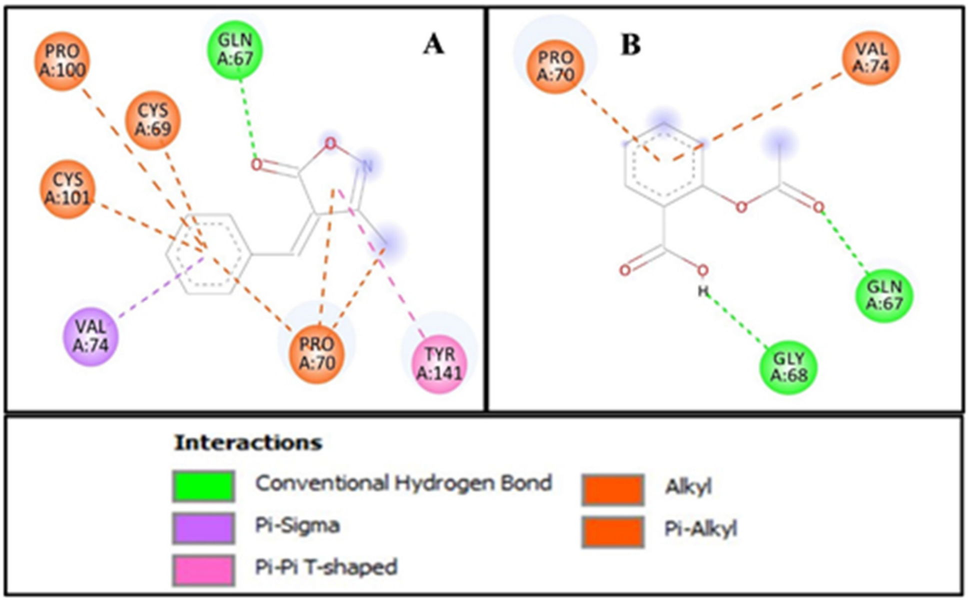

Figure S6. A and **B** represents 2D interactions of MBO and Aspirin against target: TNF-α respectively, evaluated through Biovia Discovery Studio 2016.

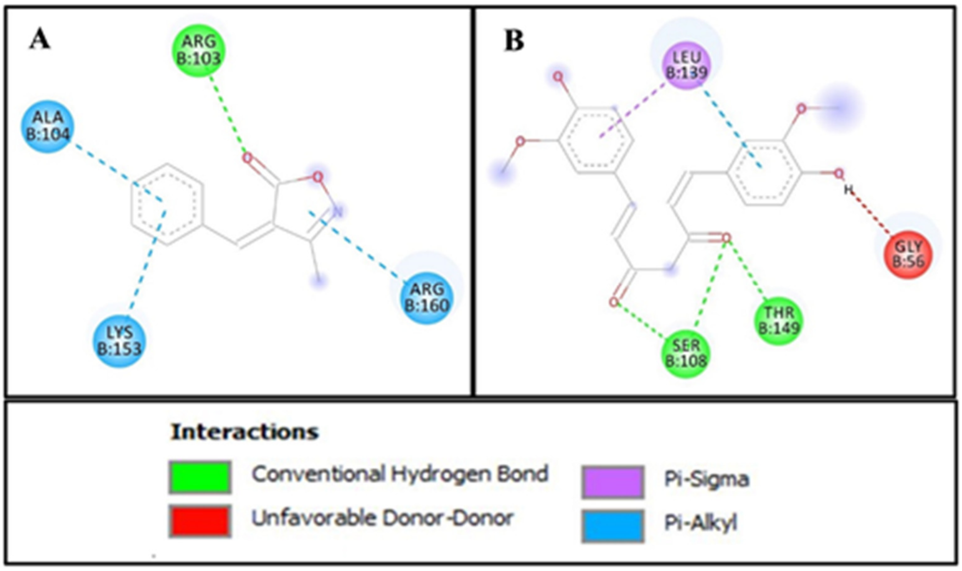

Figure S7. A and **B** represents 2D interactions of MBO and Curcumin against target: NFkB respectively, evaluated through Biovia Discovery Studio 2016.

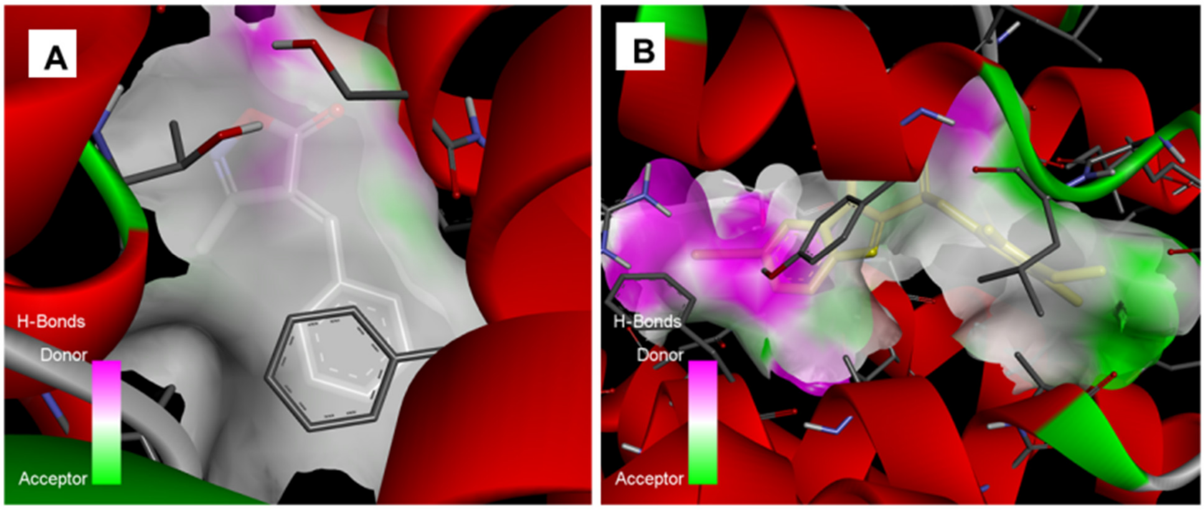

Figure S8. A and **B** represents 3D interactions of MBO and Omeprazole against target: H^+/K^+-ATPase respectively, evaluated through Biovia Discovery Studio 2016

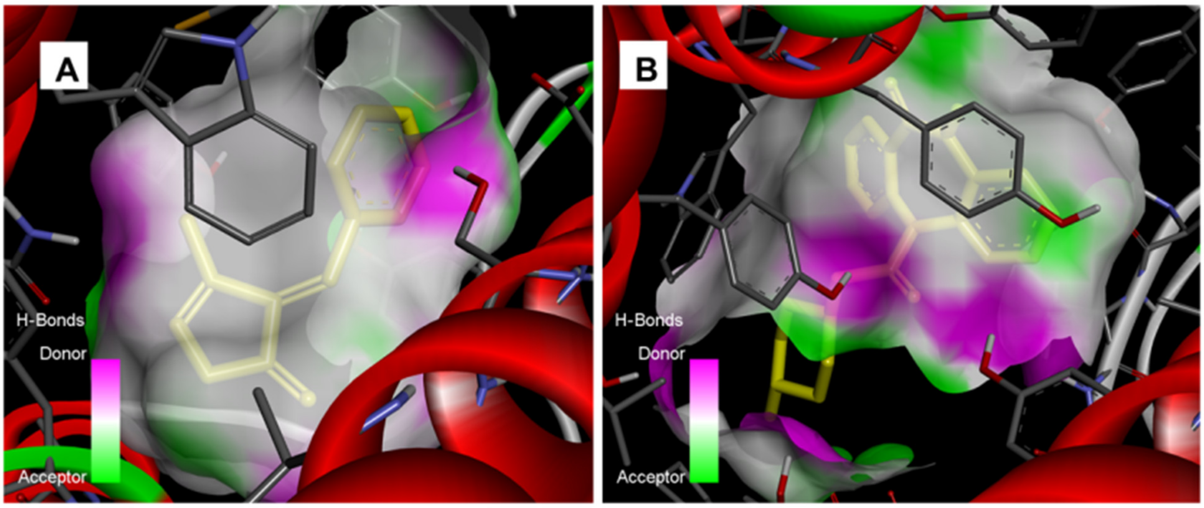

Figure S9. A and **B** represents 3D interactions of MBO and Pirenzepine against target: M1 respectively, evaluated through Biovia Discovery Studio 2016.

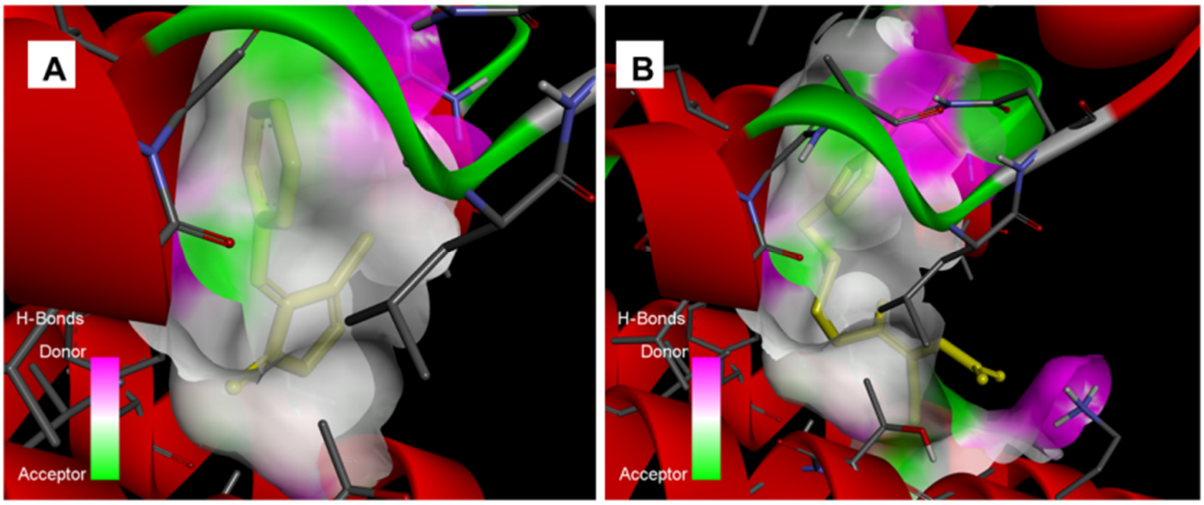

Figure S10. A and **B** represents 3D interactions of MBO and Ranitidine against target: H_2 respectively, evaluated through Biovia Discovery Studio 2016.

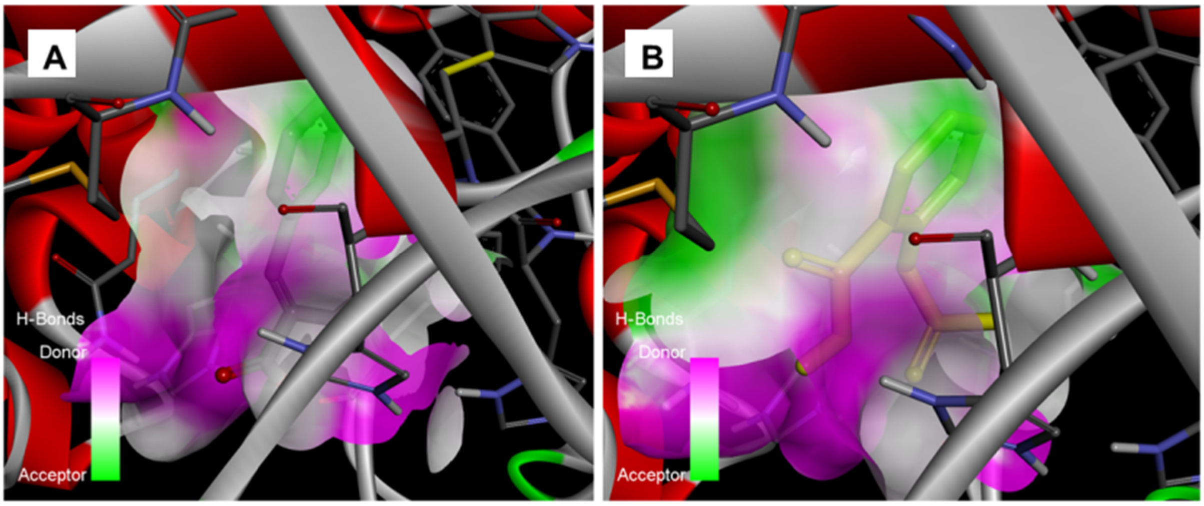

Figure S11. A and **B** represents 3D interactions of MBO and Aspirin against target: COX-1 respectively, evaluated through Biovia Discovery Studio 2016.

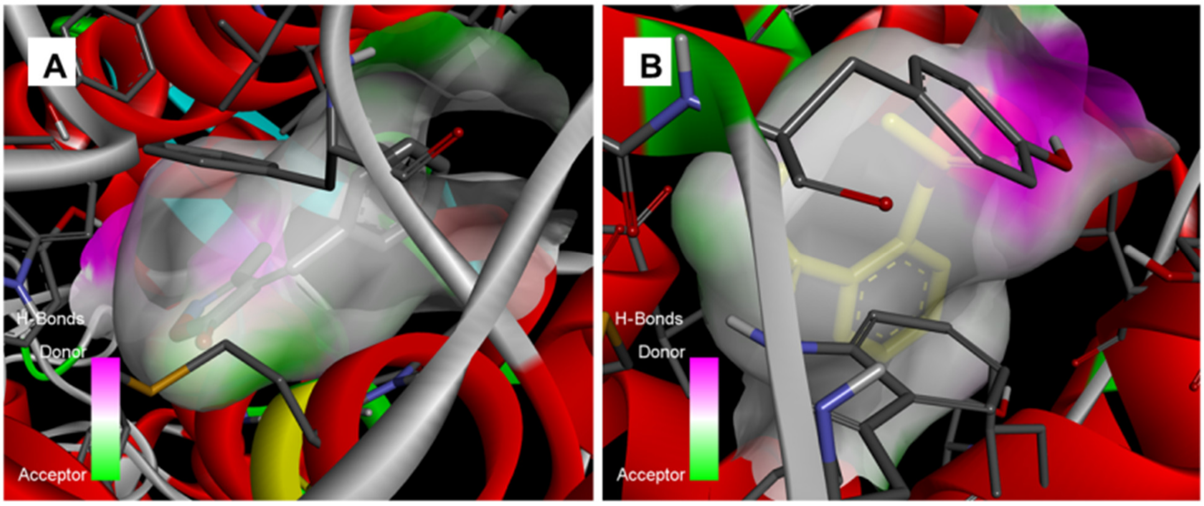

Figure S12. A and **B** represents 3D interactions of MBO and Aspirin against target: COX-2 respectively, evaluated through Biovia Discovery Studio 2016.

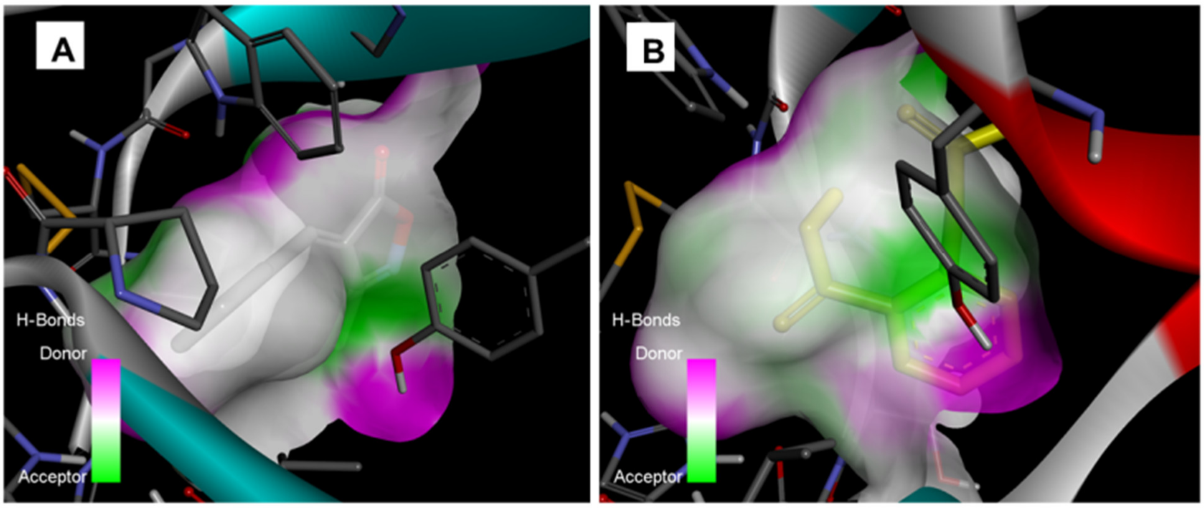

Figure S13. A and **B** represents 3D interactions of MBO and Aspirin against target: TNF-α respectively, evaluated through Biovia Discovery Studio 2016.

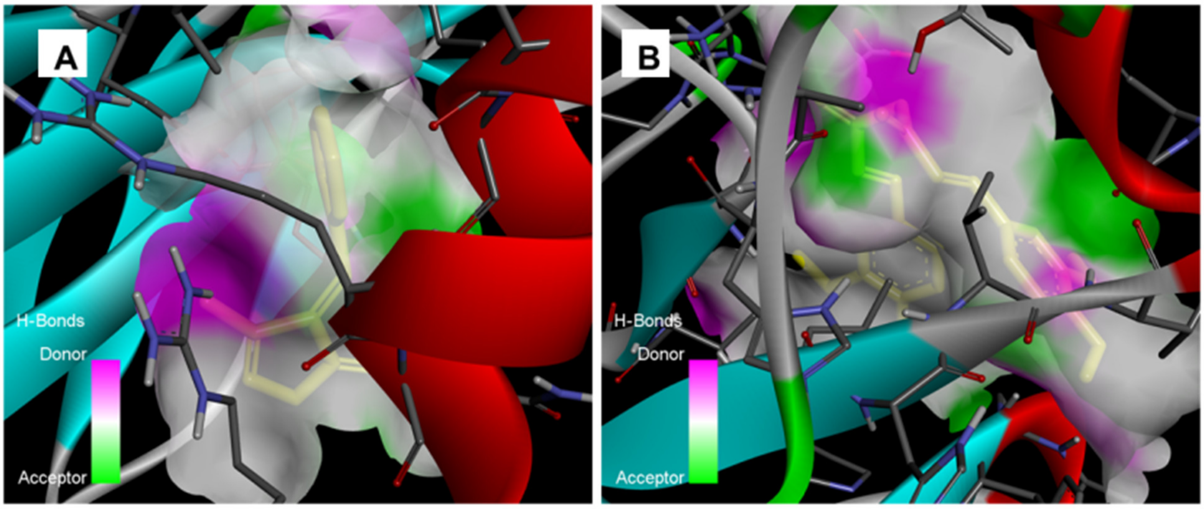

Figure S14. A and **B** represents 3D interactions of MBO and Curcumin against target: NFkB respectively, evaluated through Biovia Discovery Studio 2016.
